# Influence of Immunocastration on Slaughter Traits and Boar Taint Compounds in Pigs Originating from Three Different Terminal Sire Lines

**DOI:** 10.3390/ani11010228

**Published:** 2021-01-18

**Authors:** Ivona Djurkin Kušec, Emilija Cimerman, Martin Škrlep, Danijel Karolyi, Kristina Gvozdanović, Miodrag Komlenić, Žarko Radišić, Goran Kušec

**Affiliations:** 1 Josip Juraj Strossmayer University of Osijek, Faculty of Agrobiotechnical Sciences Osijek Vladimira Preloga 1, 31000 Osijek, Croatia; idurkin@fazos.hr (I.D.K.); kgvozdanovic@fazos.hr (K.G.); zradisic@fazos.hr (Ž.R.); 2 Directorate for Professional Support to the Development of Agriculture and Fisheries, Ministry of Agriculture, Republic of Croatia, Bani 110, 10000 Zagreb, Croatia; Emilija.Cimerman@mps.hr; 3 Agricultural Institute of Slovenia, Hacquetova Ulica 17, 1000 Ljubljana, Slovenia; martin.skrlep@kis.si; 4 University of Zagreb, Faculty of Agriculture, Svetošimunska Cesta 25, 10000 Zagreb, Croatia; dkarolyi@agr.hr; 5 Belje Plus d.d., Svetog Ivana Krstitelja 1a, 31326 Darda, Croatia; miodrag.komlenic@belje.hr

**Keywords:** pig, immunocastration, meat quality, carcass traits, androstenone, skatole

## Abstract

**Simple Summary:**

Due to the negative public opinion and welfare issues related to surgical castration, gradual introduction of alternatives like entire males and immunocastrates is taking place. Despite several economical and welfare advantages that the alternatives offer, numerous issues (i.e., boar taint, meat quality alterations), as well as their interactions with rearing and breeding (genetic) factors remain unanswered. Therefore, the focus of this study was to investigate the possibility of using different commercial sire lines in three male sex categories (entire males, immunocastrates, surgical castrates) and to compare their carcass traits, meat quality and boar taint compounds. A significant effect of terminal sire line and/or male category together with their interaction was observed for most of the investigated carcass and meat quality traits. The observed interaction should be taken into consideration when deciding on the production of a specific male category.

**Abstract:**

The aim of the research was to investigate the influence of terminal sire line (TSL) and male category (MC) on carcass and meat quality of commercial fatteners. The study was performed on 180 pigs originating from three terminal sire lines: A (Pietrain × Large White), B (pure Pietrain), and C (Pietrain × Duroc × Large White), being assigned to three groups according to MC: immunocastrates (IC, *n* = 60), surgical castrates (SC, *n* = 60) and entire males (EM, *n* = 60). TSL affected most of the carcass and meat quality traits, together with the androstenone concentration. At the same time, MC had a significant effect on fat thickness, ham circumference, drip loss, cooking loss and androstenone and skatole concentrations. A significant interaction effect was observed for carcass length and ham circumference, as well as for most of the measured meat quality traits (except cooking loss, CIE L*, CIE b*, and Warner Bratzler Shear Force (WBSF)). Among the three investigated sire lines, immunocastration was shown to be most beneficial for fatteners sired by the TSL C. However, if deciding to raise EM, fatteners from the TSL B are recommended in terms of carcass and meat quality, although strategies for avoiding boar taint in their carcasses must be taken into consideration.

## 1. Introduction

Surgical castration causes severe pain to animals, thus raising a welfare concern for the pig industry [1] and a negative public attitude towards the procedure itself when performed without anaesthesia and/or analgesia. Due to these issues, gradual switching towards different alternatives like entire males (EM) or immunocastrates (IC) is taking place in many (but mostly western) European countries [2,3]. Both alternatives demonstrate not only welfare but also economic advantages over surgical castrates (SC), including better feed conversion and higher lean deposition, thus evidently improving their cost-effectiveness [4,5]. At the same time, several quality problems are also brought forward.

In EM, the occurrence of unfavourable organoleptic pork traits, the so-called “boar taint” presents the key issue. Boar taint is described as offensive urine-, sweat- and faecal-like odour and/or taste, attributed to substances skatole and androstenone that are deposited in fat tissue of uncastrated animals [6]. Besides, EM may prove problematic in terms of more aggressive behaviour [2] and inferior meat quality accompanied by low-fat saturation (being also important in terms of consumer acceptance and processing aptitude [7]).

Few effective strategies are available in attempts to avoid the boar taint in EM. One of the possible solutions includes genomic selection [8,9], which seems possible due to the high to moderately high heritability of the androstenone and skatole concentrations [10,11,12]. However, it was shown that selection for low androstenone levels can have an adverse effect on reproductive and meat quality traits in pigs [3,13] and also does not eliminate aggressive and mounting behaviour in boars [14,15]. Different feeding strategies, aimed primarily at reducing endogenous skatole production (which, opposite to androstenone, depends on numerous factors other than genetic), could be also employed. This includes the addition of hydrolysable tannins [16,17,18], chicory [19,20,21,22] or fructooligosaccharide [23] in the pig diet. Finally, modifications of pre-slaughter conditions [24,25] and immunocastration [6] can also be used for addressing this issue.

Compared to EM, immunocastrated pigs exhibit certain advantages (especially during the last phase of growth after effective immunization), such as better feed efficiency and reduced aggressiveness [26] without significant deterioration in meat quality [27]. On the other hand, the rearing of IC has some potential weaknesses including the problems in the detection of boar taint at the slaughter line [28], the uncertainty of the producers regarding the consumer acceptance of such meat [29] and negative public attitude towards immunocastration in some countries [30].

The comparison of meat quality and carcass characteristics between different pig male categories (EM, SC and IC) has been described by numerous authors [8], but is, especially in the case of meat quality properties, still lacking consistency and needs further confirmation [31,32]. An additional aspect to consider in regard to pork quality traits is the effect of pig breed or genetic background. As revealed by the meta-analytical study of Trefan et al. [33] investigating both breed and pig sex category effect, the effect of both should be taken into consideration and is potentially offering the pork producers an opportunity to achieve desired pork quality characteristics. Still, the information on the influence of pig sex category in interaction with genotype (e.g., breeds, crossbreeds, different terminal sire lines) on productive performance and meat quality is relatively scarce and remains largely undefined [8].

Therefore, the aim of the present study was to investigate the influence of different male categories in relation to their genetic background (i.e., modern terminal sire line) on carcass traits, meat quality and boar taint compounds of fatteners originating from three different male categories reared in an intensive production system.

## 2. Materials and Methods

### 2.1. Animals

The experimental protocol was approved by the Bioethics Committee of the Faculty of Agrobiotechnical Sciences Osijek (2158-94-02-20-05, 16.102020), and all procedures were performed in accordance with the Croatian Animal Welfare Act and other legal acts regulating animal husbandry and welfare.

The study was performed on 180 fatteners originating from three different commercial terminal sire lines (TSL): A (Pietrain × Large White), B (pure Pietrain) and C (Pietrain × Duroc × Large White). The animals used in the experiment were selected from 30 litters farrowed within two weeks. The sows used were all of the identical genotype (Landrace).

Within each litter, two pigs were surgically castrated (SC; 60 animals in total), two were designated for immunocastration (IC; 60 animals in total) and two were raised as entire males (EM; 60 animals). Surgical castration was performed during the first week of life. In the IC group, pigs were vaccinated by applying two doses of Improvac^®^ (Pfizer Animal Health, Parsippany, NJ, USA), as recommended by the manufacturer. During the experiment, the animals were group-penned (10 pigs/pen) according to their designated sex category.

The size of the pens was 3.60 m × 2.40 m, allowing 0.86 m^2^ of available floor space per animal. The floor was partly concrete slatted and partly fully solid, with the fresh straw added every day to cover most of the fully solid area. In addition to natural light, artificial light was applied for 13 h per day. The temperature was 34 °C at birth and was gradually lowered to 13 °C before slaughter.

In order to avoid stress and aggressiveness, pigs were kept in the same pens from weaning to the end of the experiment. All pigs were kept under the same housing conditions with ad libitum access to food and water.

During the trial, the animals were fed the following commercial diets (Belje plus d.d., Darda, Croatia): SO-1 (13.58 MJ ME/kg and 17.36% crude protein) from weaning to approximately 30 kg live weight (LW); ST-2 (13.26 MJ ME/kg and 16.05% crude protein) from 30 to approximately 70 kg LW; and ST-3 (12.95 MJ ME/kg and 14.06% crude protein) from 70 kg LW to the end of the experiment.

### 2.2. Slaughter Procedure, Carcass Composition and Meat Quality Traits

After reaching the average weight of 110 kg, the animals were transported to a nearby commercial abattoir where they were slaughtered according to the conventional procedure that included stunning with CO_2_.

After 24 h of cooling, the carcass length (distance from the cranial edge of *os pubis* to cranial edge of 1st rib), ham length (from the anterior edge of the symphysis *ossis pubis* to the hock joint) and the ham circumference at its widest point were measured. Lean meat percentage (LMP), muscle thickness (M) and fat thickness (S) were determined by the two-point method approved in Croatia [34].

Initial (pH_45_) and final (pH_24_) pH values were measured 45 min and 24 h post mortem in *longissimus dorsi* (LD) muscle using Mettler MP 120-B portable pH meter (Mettler-Toledo, Schwerzenbach, Switzerland). Drip loss was determined using the EZ-DripLoss method [35]. For objective colour evaluation (CIE L*a*b*), the average of three measurements was taken using Minolta CR-410 colorimeter (Minolta Camera Co. Ltd., Osaka, Japan), calibrated against a white plate with a D65 light source and a 2° standard observer angle. Instrumental tenderness was measured on 2.54 cm thick loin chops that were frozen for two weeks, defrosted for 24 h at 4 °C overnight, cooked in a water bath to 73 °C internal temperature and cooled at 4 °C overnight. Shear force was measured on at least six 1.27 mm-thick cores using TA.XTplus Texture Analyser (Stable Micro Systems, London, UK) fitted with a 1-mm-thick Warner-Bratzler shear attachment and presented as Warner–Bratzler Shear Force. Cooking loss was calculated as a percentage of water loss during cooking the samples for the instrumental tenderness assessment.

### 2.3. Boar Taint Compounds

Androstenone and skatole levels were determined on subcutaneous fat tissue taken at the level of the last rib from IC and EM carcasses; elevated levels of boar taint substances in SC animals were not expected, and therefore this group was excluded from the quantification of androstenone and skatole. Quantitative analyses of androstenone and skatole concentrations were determined by HPLC as described by Hansen-Møller [36] and Pauly et al. [37] and expressed as µg/g of liquid fat. The detection limits were 0.24 µg/g for androstenone and 0.03 µg/g for skatole.

### 2.4. Olfactory Analysis of the Boar Taint

The intensity of the boar taint was assessed in IC and EM by a trained six-member sensory panel based on ISO 4121 (Sensory analysis—Guidelines for the use of quantitative response scales; https://www.iso.org/standard/33817.html) and ISO 13299 (Sensory analysis—Methodology—General guidance for establishing a sensory profile; https://www.iso.org/standard/58042.html). All panellists were able to detect both androstenone in the pure form and preselected tainted samples of meat.

The samples (108 in total) were taken from head meat and salivary glands according to German directive AVVLmHyg (2011) [38] and were heated using the boiling method and the microwave method as described by Whittington et al. (2011) [39]. According to the levels of boar taint odour intensity, samples were scored as none, mild or strong. All samples were individually assessed by each of the panellists.

### 2.5. Statistical Analysis

The obtained data were analysed by factorial ANOVA (3 × 3 factorial design) with log transformation of androstenone and skatole concentrations prior to the analyses. The influence of the TSL, male category (MC) and their interaction (TSL * MC) was determined by the Tukey HSD test, where *p* < 0.05 was classified as a significant difference. The obtained data were analysed using the Dell Statistica (2015) [40], while the incidence of the boar taint odour together with confidence intervals of 95% was calculated using the DescTools package [41] in the R environment [42].

## 3. Results and Discussion

### 3.1. Carcass Traits

TSL influenced (*p* < 0.01) several carcass traits of investigated pigs, except carcass weight, muscle thickness and ham length (Table 1). Pigs originating from the boars of TSL C had higher fat thickness (F, *p* < 0.01) than those originating from TSL A and lower LMP *(p* < 0.01) than fatteners originating from both TSL A and B. In addition, the progeny of TSL C sires had significantly longer carcasses compared to progeny of the TSL A (*p* < 0.01). Progenies of the TSL B did not differ with the other two lines in this trait. The hams of pigs originating from TSL A had significantly lower circumference than the pigs from TSL B and C (*p* < 0.01). The results of the current study regarding the backfat thickness and LMP support the earlier findings of Škrlep et al. [43], Edwards et al. [44], Latorre et al. [45], Radović et al. [46], and Vidović et al. [47]. These authors found lower backfat thickness and high LMP in Pietrain sired fatteners, whilst the inclusion of other breeds into the genetic line of sires was reported to deteriorate these traits. The fatteners sired by Duroc boars had longer carcasses with higher ham circumference, but also thicker backfat. On the other hand, Kušec et al. (2004) [48] reported a higher carcass length in crossbred pigs sired by Pietrain boars.

A significant effect of MC was observed for fat depth (*p* < 0.001) and ham circumference *(p* = 0.016). The highest fatness was recorded in SC pigs followed by IC, and EM pigs had the lowest (*p* < 0.01). Similar results were also reported by a number of other similar studies [49,50], agreeing also with the meta-analyses of Trefan et al. [33], Batorek et al. [51] and Nautrup et al. [52]. Muscle thickness (M) was not influenced by MC (*p* = 0.473), agreeing with the results of Škrlep et al. (2010 and 2011) [43,49], and also Škrlep et al. (2020) [53], who did not find differences in most of the muscle traits between different male categories, and indicated that castration (including immunocastration) may not be related to notable differences in muscle growth and that it affects mostly fat deposition.

In the present study, no significant differences were detected in LMP between the MC of investigated pigs, which is not in line with the literature [33,51], indicating SC as having the lowest, IC the intermediate, and EM the highest LMP. Nevertheless, LMP is calculated based on both carcass muscle and fat thickness, which may have counteracted in the present study.

The carcasses of investigated male categories did not differ in their length. These results are also in disagreement with the findings of other studies where EM and IC pigs exhibited longer carcasses than SC [54,55]. It should be noted that the nutritional requirements of different MC are not the same, which can significantly affect carcass traits [56].

**Figure 1:**
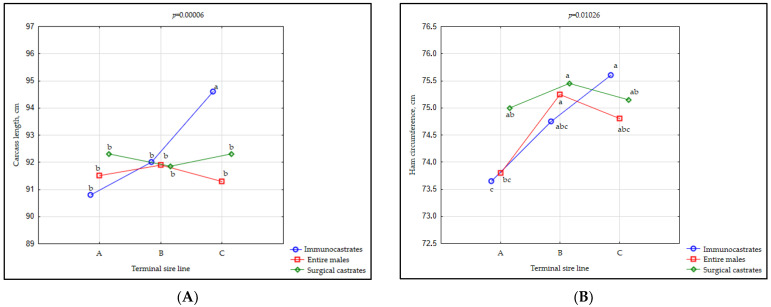
(**A**) Terminal sire line (TSL) by male category (MC) interaction for carcass length (*os pubis*—1st rib) and ham circumference (**B**). Terminal sire line A = Pietrain × Large White; Terminal sire line B = pure Pietrain; Terminal sire line C= Pietrain × Duroc × Large White. Significant differences (*p* < 0.05) between groups are denoted by different lowercase letters (a, b, c).

A significant interaction of TSL and MC was observed for carcass length (*p* < 0.001) and ham circumference (*p* = 0.010), as presented in Figure 1A,B.

The highest values recorded for carcass length were found in the IC pigs that originated from TSL C. These were significantly different from carcass lengths found in IC pigs sired by TSL A and B as well as in EM and SC pigs originating from all sire lines involved in the study (Figure 1A; Table S1). Within the pigs originating from TSL A and B as well as between them, no statistically significant differences were found in carcass length. The lowest ham circumference was found in the IC pigs sired by terminal line A boars and significantly differed from all SC pigs, as well as from EM originating from TSL B. Among IC animals, the highest ham circumference was found in pigs originating from the terminal sire C, and among EM from terminal sire line B, although it did not statistically differ from EM sired by C boar. In SC animals, the highest ham circumference was found in fatteners originating from the TSL B, although SC animals did not differ in this trait (Figure 1B; Supplementary Table S1).

The highest length and ham circumference that was found in IC pigs from TSL C (25% Pietrain and 25% Duroc) is not easy to explain. There are not many studies dealing with the interaction between the male category and breed on carcass traits [3]. As to the diverse differential effect of ST in regard to the pig progeny, the existing literature mainly reports no significant or very limited effect [57,58,59]. Contrary to the results of the present study, Škrlep et al. (2011) [43] found no interaction in the experiment with two crossbreds (50% Duroc and 50% Pietrain) and three treatments (IM, IC, EM). Nonetheless, the authors found IC pigs to be superior compared with SC pigs in terms of carcass properties, which was not confirmed in the present study.

Generally, crosses sired with Pietrain are superior in lean meat percentage (LMP) and its indicators (fat depth and muscle thickness), whilst Duroc sires produce progeny characterized by superior growth rate, but with lower LMP in the carcasses. Due to higher intramuscular fat (IMF), their meat is therefore more appropriate for the production of high-quality products like dry-cured hams [45,60,61]. The situation regarding certain carcass traits such as carcass or ham length is, however, less clear. For example, Edwards et al. (2003) [44] reported longer carcasses in Duroc sired pigs than in the progeny of Pietrain boars, whilst Kušec et al. (2004) [48] found reverse results. Morales et al. (2013) [62] discussed that such inconsistencies could occur due to the differences in the genetic potential of Duroc and Pietrain lines used in the investigations. Indeed, Latorre (2003a) [63] and Nguyen and McPhee (2005) [64] suggested that variability in carcass traits among lines within the breeds can be higher than the variability between the breeds. In the present study, IC pigs originating from TSL C, the only boars having 50% Duroc in genetic setup, had the longest carcasses and the widest hams.

### 3.2. Meat Quality Traits

**Figure 2:**
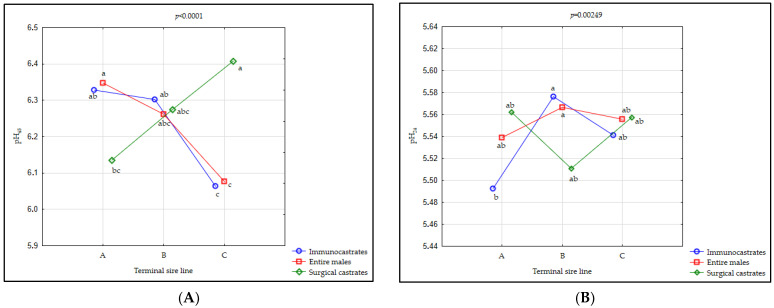
The interaction between immunocastrates (IC), entire males (EM) and surgical castrates (SC) originating from different terminal sire lines. (**A**) and pH_24_ in LD muscle (**B**). Terminal sire line A = Pietrain × Large White; Terminal sire line B = pure Pietrain; Terminal sire line C= Pietrain × Duroc × Large White. Significant differences (*p* < 0.05) between groups are denoted by different lowercase letters (a, b, c).

The effect of TSL and MC on meat quality traits is given in Table 2. Significant differences (*p* < 0.05) between different sire lines in pH_45_ were observed (i.e., the highest values in C and the lowest in B); however, no differences could be found between different male categories for this trait. In agreement with the results of this study, the results of the three meta-analyses showed no or very small difference between different male categories in the case of muscle pH, indicating this trait to be without actual practical importance in regard to sex categories [7,26,32,65]. We also found a significant interaction (*p* < 0.001) between the TSL*MC on initial pH values (Figure 2A; Table S1). Surgical castrates originating from TSL C and EM originating from TSL A exhibited the highest pH_45_, while the lowest value for this trait was found in IC and EM animals originating from the C TSL (Table S1; Figure 2A). However, although significant differences between different MC and different TSL were observed, this difference may probably have no practical meaning, as observed values were above 5.8 [66], indicating a normal rate of pH decline in all investigated pig groups.

Although no significant differences (*p* > 0.05) were found between different genotypes and male categories for pH_24_ (Table 2), a significant interaction (*p* = 0.006) between TSL and MC was observed (Table S1, Figure 2B). The highest pH_24_ was observed in IC from the TSL B, indicating a positive influence of immunocastration on this trait in progenies of this sire line. On the other hand, the pH_24_ of the IC originating from the TSL A was even lower than the threshold value for “normal” meat (pH_24_ > 5.5), as suggested by Forrest [67]. Contrary to our results, in studies on crossbreds with 25 or 50% of Pietrain genetics [58,68] and different percentages of Duroc in Large White breed [57], no interaction between male category and genotype was observed for this trait.

It is known that water holding capacity strongly influences pork sensory traits, especially colour, flavour, and tenderness, apart from its economical and nutritional influence [69]. In the present study a significant effect (*p* < 0.001) of TSL (higher in C compared to A and B), and (*p* = 0.031; higher in EM compared to SC), as well as their interaction (*p* = 0.002) on drip loss values was observed.

**Figure 3:**
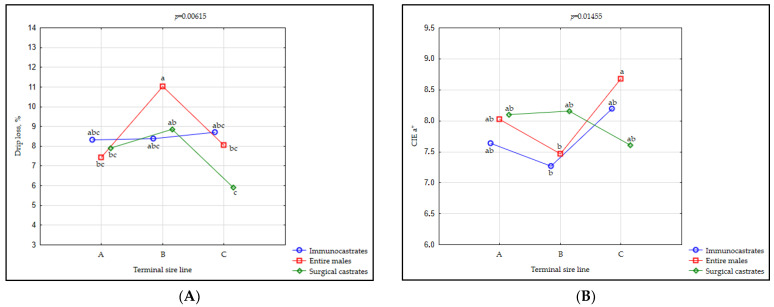
The interaction plot between terminal sire line (A, B, C) and male category (IC = immunocastrates; EM = entire males; SC = surgical castrates) for drip loss (**A**) and CIE a* values (**B**). TSL A = Pietrain × Large White, TSL B = pure Pietrain, TSL C= Pietrain × Duroc × Large White. Significant differences (*p* < 0.05) between groups are denoted by different lowercase letters (a, b, c).

EM originating from the TSL B exhibited the highest drip loss, while the lowest drip loss was observed in SC animals originating from the TSL C. Within TSL A, IC exhibited higher drip loss values than EM and SC animals, although no significant differences were observed between different male categories in this trait. In TSL C, SC exhibited the lowest drip values, followed by EM and IC animals (Table S1; Figure 3A).

In their review on meat quality of different male sex categories, Škrlep et al. (2020) [7] argued that the most probable cause of the inferior water holding capacity usually found in EM and IC animals is the elevated muscle protein oxidation, which, coupled with oxidation of unsaturated fats, causes denaturation, loss of solubility and myofibrillar shrinkage and thus reduces the ability of the muscle to bind water. Corroborating the fact that lower carcass fatness also means lower fat saturation [70], this could explain MC-related differences (i.e., higher drip loss observed in EM). Similar to the result for animals originating from TSL B, in the investigation of Škrlep et al. (2011) [43] on two types of crossbreds (Duroc and Pietrain sired), drip loss measured after 24 h and 48 h was also influenced by sex and crossbreed type interaction but without any differences between the MCs. The results of individual studies investigating only the sex type as an influential factor are ambiguous; however, generally, no difference between different sex types was found for this trait [7,32,49]. It should be noticed that observed drip loss was quite high in all investigated groups; however, this may be more related to the relatively low age of the animals, especially for entire males, which still did not manage to exhibit their full physiological potential at this stage of growth and development [71,72].

Similar to drip loss, the cooking loss measured after cooking the sample for shear force measurement, was influenced by MC (*p* < 0.001), with EM having the highest values for this trait (Table 2). These results are in concordance with those reported by Škrlep et al. (2020) [53]. In the same study, it was also reported that EM had higher WBSF values than IC and SC. The results of meta-analytical and several latter studies generally indicate EM as exhibiting the toughest meat [7]. However, we did not find significant differences between different MCs for this trait. On the other hand, the shear force was influenced by the TSL (*p* = 0.013), where meat from fatteners originating from TSL B was tenderer than the meat from fatteners originating from TSL A. The difference is difficult to relate to any of the measured meat quality or carcass traits as influenced by TSL. It is generally accepted that pigs with higher leaner growth efficiency also exhibit tougher meat [73]. It should be noted, however, that performance of the particular sire progeny for this trait is also dependent on the dam line used [74] and therefore is more a contribution of a particular sire × dam combination rather than the terminal sire line alone. Furthermore, as can be observed from Table 2, the absolute difference between the three TSLs is rather low and does not have much practical importance.

Pigs originating from the TSL B had meat with a more pronounced red colour (CIE a*) compared to pigs originating from the TSL C (Table 2). Although no significant difference was found between different MC, it can be observed (Figure 3B and Supplementary Table S1, depicting interaction in the case of this trait (*p* = 0.009)) that in TSL C, EM produced meat with a more pronounced red colour than entire males (line B) or immunocastrates (line B). Similar results were reported by Gispert et al. (2010) [54] in (Landrace × Duroc) × Pietrain pigs. Contrary to the results of the current study, Škrlep et al. (2020) [53] observed the highest CIE a* values in IC animals of the same crossbreds, whereas another study by Li et al. (2015) [68] found no significant influence of interaction between male category and genotype in 25 and 50% Pietrain-sired fatteners.

### 3.3. Androstenone and Skatole

The mean concentrations of androstenone and skatole in backfat between TSL and different MC, as well as the significance of both factors and their interactions, are shown in Table 3.

The concentration of androstenone was significantly influenced by both tested factors (with significant interaction; *p* < 0.001), while the concentration of skatole was influenced only by the MC (*p* = 0.0039). With regard to the TSL, the highest backfat mean androstenone level was demonstrated in EM and IC pigs from the TSL C (in average 1.51 µg per g of fat), while pigs from other two sire lines had similar and significantly lower androstenone levels (0.68 and 0.73 µg/g in A and B, respectively; Table 3). The age of reaching puberty (sexual maturity) in EM as well as the androstenone and skatole levels in fat at slaughter may differ between breeds [75,76]. Among breeds, Duroc has been known for its higher androstenone levels in fat compared to other breeds [77], including Pietrain and Large white [78]. Duroc sired pigs also generally have a higher growth rate than Pietrain sired pigs [45,79]. Hence, it can be assumed that such a high concentration of androstenone found in the backfat of pigs from TSL C (well above the sensory threshold detection level for androstenone of 1 μg/g) are, at least partly, a consequence of the earlier entry into sexual maturity characterised by Duroc breed. However, one should keep in mind that sexual maturity was not tested prior to the sampling for androstenone and skatole concentrations.

The differences in hepatic metabolism and metabolic clearance of androstenone from fat tissue that exists between breeds [80,81] may also contribute to the present result.

The concentration of skatole observed in the present study was generally below the sensory threshold detection level (0.2 μg/g), regardless of the TSL. It is known that marked differences in skatole levels in plasma and fat may exist between breeds [82] and higher skatole levels in the backfat of EM have been associated with the Large White breed in some studies [83]. In the present work, pigs from the TSL A with the highest share of the Large White genetics indeed showed the higher skatole level in backfat than pigs with lower (TSL C) or no (TSL B) inclusion of this breed. However, this result did not fully corroborate the previous findings as observed differences in skatole concentrations in fat tissue between sire lines did not reach statistical significance.

With regard to MC, as expected, EM had significantly higher levels of androstenone (1.66 µg/g) and skatole (0.092 µg/g) in back fat compared to IC (0.28 µg/g and 0.033 µg/g, respectively). The present results are consistent with the previous research studies that showed an effective reduction of compounds responsible for boar taint after effective immunocastration [31,49,84].

The proportion of the tainted samples (according to threshold values proposed by Walstra et al. (1999) [85] is presented in Table 4. It can be observed that in all IC animals, there was only one animal originating from TSL B that exhibited androstenone levels above >1.0 µg/g, which indicates that vaccination against gonadotropin-releasing hormone (GnRH) generally was successful. Additionally, in the same male category, no animals exhibited skatole level above 0.25 0 µg/g.

On the other hand, 79% of the entire males originating from TSL C, 40% of the pigs sired by TSL B boar and 35% of EM originating from TSL A exhibited androstenone levels above 1.00 µg/g. Despite the high incidence of elevated androstenone level, in TSL B and C, only 5% of the animals (i.e., one animal) had skatole concentration above 0.25 µg/g, and in EM originating from TSL A, skatole levels above the designated threshold were observed in 10% of the animals. It should be noted, however, that in the majority of animals with elevated androstenone/skatole levels, their values were close to the threshold.

**Figure 4:**
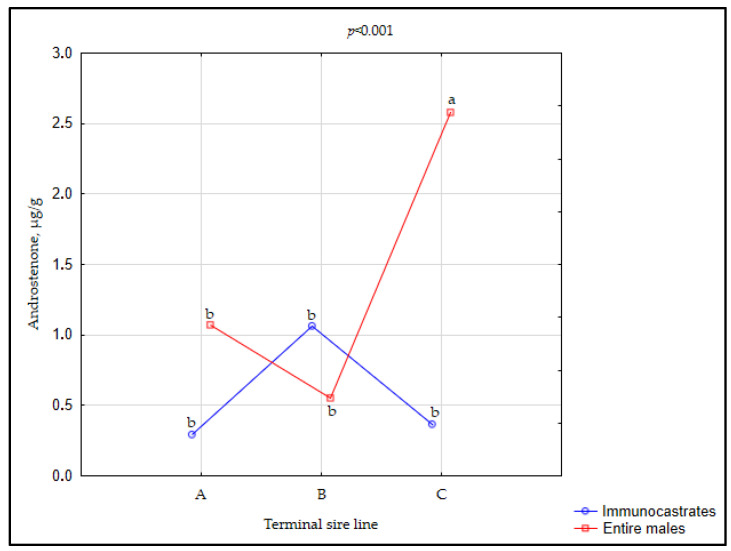
Interaction between terminal sire line and male category (MC) for androstenone concentration in immunocastrates (IC) and entire males (EM). TSL A = Pietrain × Large White, TSL B = pure Pietrain, TSL C= Pietrain × Duroc × Large White. Significant differences (*p* < 0.05) between groups are denoted by different lowercase letters (a, b, c).

A significant interaction between MC and TSL existed for the androstenone level (Figure 4). EM from TSL C had higher androstenone levels than entire males from the other two sire lines, but such breed dependence was not observed among IC animals.

### 3.4. Olfactory Analysis

**Figure 5:**
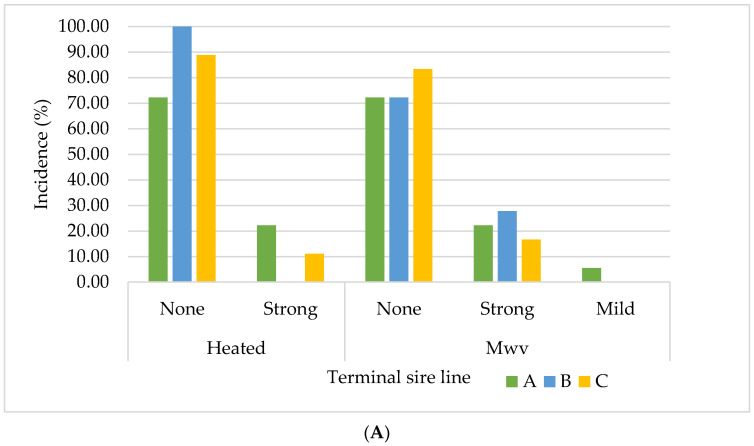

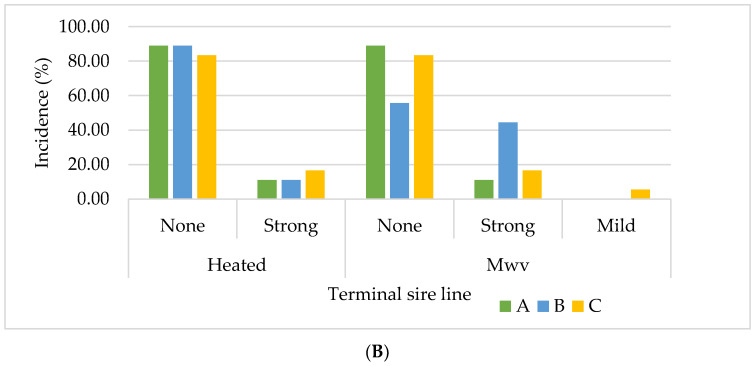
The incidence of boar taint odour of the (**A**) immunocastrates and (**B**) entire males samples originating from three terminal sire lines using hot water (heated) and microwave (mwv) method.

The results of the olfactory analysis together with confidence intervals for incidence of boar taint in EM and IC samples from three TSL are presented in Figure 5 and Table S2. A discrepancy between the results objectively determined by HPLC device and sensory analysis, as well as between two olfactory methods (hot water and microwave), can be observed. Although in IC category only 5% of the animals had androstenone levels above 1.00 µg/g and none of the animals exhibited skatole concentration above 0.25 µg/g, the sensory panel scored approximately 11% of the samples as having mild/strong boar taint odour when using the hot water method. This frequency increased when using the Mwv method to as high as 40% in TSL B. This is, however, no surprise, because the assesment of the boar taint intensity largely depends on the sensitivity of the assesor [86,87], due to which the true threshold for sensory evaluation of androstenone and skatole levels still does not exist [88].

Additionally, the disagreement between the sensory scores of the two different olfactory methods was also observed (Figure 5). For instance, when cooked in water, 100% of the samples from IC originating from TSL B were classified into the group with characteristic pork odour; however, when heated in the microwave, 27.78% of the samples were classified into the tainted group. The observed discrepancy is in agreement with the results of Trautmann et al. (2016) [89], who argued that this is a consequence of the heating method prior to sensory evaluation. By comparing three available sensory methods (hot water, microwave, hot iron) the authors found that the probability of individual false positive ratings is significantly lower for hot water and hot iron than for the microwave method.

Interestingly, although most of the tainted samples identified by the sensory panel originated from the TSL B, samples originating from the TSL C were those with the highest concentration of both boar taint compounds as determined by chemical analysis (79% vs. 35% for line A and 40% for line B). Unlike in samples from TSL A and B, with elevated levels of androstenone together with skatole concentration above 0.1 μg/g (data not shown), those samples had high androstenone levels, but their skatole levels were all lower than 0.1 μg/g fat. The research on the tainted patties showed that androstenone levels up to 2.07 μg/g in back fat are acceptable if skatole levels are low (≤0.10 μg/g), while skatole levels in back fat higher than 0.10 μg/g are not acceptable at any androstenone level [88]. Low skatole levels observed in fatteners originating from TSL C are the most probable cause of their better score according to the sensory panel. This confirms that sensory perception of off-odours in fat tissue develops by the interaction of androstenone and skatole, with skatole being more important for boar-taint perception [88] and that, generally, the preference of the boar meat decreases with the increase in skatole and androstenone, although the meat or meat product is more disliked when containing higher skatole levels [88,89,90].

## 4. Conclusions

The results of this research showed that interaction between TSL and MC plays a significant role in carcass and meat quality traits in modern fatteners and therefore should be taken into consideration when deciding on the production of a specific male category. The immunocastration proved to be most beneficial for the fatteners originating from TSL C, with a positive influence on carcass traits and without significant deterioration of meat quality. Terminal sire line B (regardless of the MC) produced fatteners with higher lean meat percentage and lower drip loss and WSBF values, with a more pronounced red colour of the meat. The EM from this TSL had longer carcasses and higher ham circumference, with similar meat quality traits compared to EM from the other two TSL. On the other hand, due to somewhat higher skatole levels (>0.1 μg/g), more IC and EM carcasses were scored as tainted, due to which precaution measures for avoiding boar taint should be implemented. As for the SC, the best performance in carcass traits was observed for animals sired by TSL B sire; however, SC from the TSL C exhibited to some extent better meat quality.

Sensory analysis revealed the discrepancy between the used methods, with the microwave method classifying more samples into the tainted group. Due to observed discrepancy and the lack of threshold values in sensory evaluation, classifying the carcasses according to objective measurements of androstenone and skatole concentrations is still recommended.

## Figures and Tables

**Table 1:** Effect of terminal sire line and male category (LS mean ± SEM) on carcass traits.

Trait	Carcass Weight, kg	F, mm	M, mm	LMP, %	Carcass Length, cm	Ham Length, cm	Ham Circumference, cm
Terminal sire line (TSL)							
A	95.1 ± 0.35	15.8 ^a^ ± 0.54	72.6 ± 0.69	57.9 ^a^ ± 0.44	91.5 ^b^ ± 0.28	34.3 ± 0.14	74.2 ^b^ ± 0.15
B	95.3 ± 0.41	15.1 ^b^ ± 0.44	71.8 ± 0.64	57.7 ^a^ ± 0.52	91.9 ^ab^ ± 0.25	34.2 ± 0.10	75.2 ^a^ ± 0.15
C	96.6 ± 0.61	17.4 ^a^ ± 0.56	71.4 ± 0.63	55.6 ^b^ ± 0.43	92.7 ^a^ ± 0.33	34.6 ± 0.14	75.2 ^a^ ± 0.20
Male category (MC)							
IC	94.8 ± 0.51	16.1 ^b^ ± 0.47	71.9 ± 0.72	56.6 ± 0.43	92.3 ± 0.32	34.4 ± 0.14	74.7 ^b^ ± 0.17
EM	96.6 ± 0.49	13.5 ^c^ ± 0.37	71.4 ± 0.60	57.4 ± 0.50	91.7 ± 0.26	34.3 ± 0.12	74.6 ^b^ ± 0.20
SC	95.6 ± 0.41	18.8 ^a^ ± 0.51	72.5 ± 0.63	57.2 ± 0.51	92.2 ± 0.30	34.4 ± 0.13	75.2 ^a^ ± 0.16

SEM—standard error of the mean; TSL A = Pietrain × Large White, TSL B = pure Pietrain, TSL C= Pietrain × Duroc × Large White; F—fat depth; M—muscle thickness; LMP—lean meat percentage; SEM—standard error of mean; IC—immunocastrates; EM—entire males; SC—surgical castrates; ^a,b,c^—LS-means with different superscripts within a column and within terminal sire line or male category differ at *p* < 0.05.

**Table 2:** Effect of terminal sire line and male category (LS mean ± SEM) on meat quality traits of investigated pig groups.

Trait	pH_45_	pH_24_	Drip Loss, %	Cooking Loss, %	WBSF, N	CIE-L*	CIE-a*	CIE-b*
Terminal sire line (TSL)								
A	6.27 ^ab^ ± 0.03	5.53 ± 0.01	7.9 ^b^ ± 0.35	34.70 ± 0.18	46.90 ^a^ ± 0.76	53.30 ± 0.30	7.90 ^ab^ ± 0.17	3.70 ± 0.13
B	6.18 ^b^ ± 0.03	5.55 ± 0.01	7.6 ^b^ ± 0.41	34.40 ± 0.20	43.40 ^b^ ± 0.89	53.30 ± 0.29	8.20 ^a^ ± 0.17	3.50 ± 0.11
C	6.28 ^a^ ± 0.03	5.55 ± 0.01	9.30 ^a^ ± 0.38	34.4 ± 0.23	44.50 ^ab^ ± 0.88	53.80 ± 0.28	7.60 ^b^ ± 0.14	3.50 ± 0.12
Male category (MC)								
IC	6.26 ± 0.03	5.53 ± 0.01	8.40 ^ab^ ± 0.36	34.9 ^ab^ ± 0.17	45.80 ± 0.91	53.60 ± 0.23	7.80 ± 0.14	3.50 ± 0.09
EM	6.20 ± 0.03	5.56 ± 0.01	8.90 ^a^ ± 0.45	35.3 ^a^ ± 0.16	44.50 ± 0.89	53.10 ± 0.21	8.00 ± 0.16	3.60 ± 0.10
SC	6.27 ± 0.03	5.54 ± 0.01	7.60 ^b^ ± 0.35	33.3 ^b^ ± 0.19	44.50 ± 0.78	53.80 ± 0.29	8.00 ± 0.18	3.50 ± 0.16

* SEM—standard error of the mean; TSL A = Pietrain × Large White, TSL B = pure Pietrain, TSL C= Pietrain × Duroc × Large White; SEM—standard error of mean; IC—immunocastrates; EM—entire males; SC—surgical castrates; WBSF—Warner Bratzler Shear Force; ^a,b^—letters with different superscripts within the same column differ at *p* < 0.05.

**Table 3:** Effect of terminal sire line and male category (LS means ± SEM) on androstenone and skatole concentrations.

Terminal Sire Line (TSL)	Androstenone (µg/g)	Skatole (µg/g)
A	0.68 ^b^ ± 0.08	0.085 ± 0.02
B	0.73 ^b^ ± 0.30	0.051 ± 0.01
C	1.51 ^a^ ± 0.13	0.052 ± 0.02
Male category (MC)		
IC	0.28 ^b^ ± 0.03	0.033 ^b^ ± 0.00
EM	1.66 ^a^ ± 0.02	0.092 ^a^ ± 0.02

SEM—standard error of the mean; TSL A = Pietrain × Large White, TSL B = pure Pietrain, TSL C= Pietrain × Duroc × Large White; IC—immunocastrates; EM—entire males; ^a,b^—letters with different superscripts within a column differ at *p* < 0.05.

**Table 4:** The proportion of the “tainted” samples in different male categories according to terminal sire line.

Terminal Sire Line	Male Category	Proportion (%) of Carcasses with Skatole > 0.25 µg/g, [CI]	Proportion (%) of Carcasses with Androstenone > 1.0 µg/g, [CI]
A	IC	0 [0.00, 1.00]	0 [0.00, 1.00]
	EM	10 [0.05, 1.00]	35 [0.20, 0.88]
B	IC	0 [0.00, 1.00]	5 [0.00, 1.00]
	EM	5 [0.00, 1.00]	40 [0.25, 0.85]
C	IC	0 [0.00, 1.00]	0 [0.00, 1.00]
	EM	5 [0.00, 1.00]	79 [0.60, 0.94]

TSL A = Pietrain × Large White, TSL B = pure Pietrain, TSL C= Pietrain × Duroc × Large White; IC—immunocastrates; EM—entire males; [CI] = 95% confidence interval.

## Data Availability

The data presented in this study are available on request from the corresponding author.

## Supplementary Materials

The following are available online at https://www.mdpi.com/2076-2615/11/1/228/s1, Table S1: Significant male type x terminal sire line interaction of investigated traits. Table S2: Confidence intervals for the incidence of boar taint in different meat samples.
