# Supplementary material for: Influence of Immunocastration on Slaughter Traits and Boar Taint Compounds in Pigs Originating from Three Different Terminal Sire Lines
Source: Animals (Basel). 2021 Jan 18;11(1):228. doi: 10.3390/ani11010228 (PMC7831900; doi:10.3390/ani11010228)
Supplement: Supplementary file 1 [file animals-11-00228-s001.pdf]

animals

MDPI

Supplementary Materials

Influence of Immunocastration on Slaughter Traits and Boar Taint Compounds in Pigs Originating from Three Different Terminal Sire Lines

Ivona Djurkin Kušec, Emilija Cimerman, Martin Škrlep, Danijel Karolyi, Kristina Gvozdanović, Miodrag Komlenić, Žarko Radišić and Goran Kušec

Table S1. Significant male type x terminal sire line interaction of investigated traits.

Male type	Terminal sire line (TSL)	Carcass traits		Meat quality traits				Androstenone concentration, µg/g
		Carcass length, cm	Ham circumference, cm	pH_{45}	pH_{24}	Drip Loss, %	CIE a *	
Immunocastrates	A	90.8 [b]	73.65 [c]	6.33 [ab]	5.49 [b]	8.33 [abc]	7.64 [ab]	0.22 [b]
	B	92.0 [b]	74.75 [abc]	6.30 [ab]	5.58 [a]	8.39 [abc]	7.27 [b]	0.84 [b]
	C	94.6 [a]	75.60 [a]	6.06 [c]	5.54 [ab]	8.71 [abc]	8.20 [ab]	0.63 [b]
Entire males	A	91.5 [b]	73.80 [bc]	6.35 [a]	5.54 [ab]	7.44 [bc]	8.03 [ab]	0.79 [b]
	B	92.0 [b]	75.25 [a]	6.26 [abc]	5.57 [a]	11.03 [a]	7.47 [b]	0.53 [b]
	C	91.3 [b]	74.80 [abc]	6.08 [c]	5.56 [ab]	8.05 [bc]	8.67 [a]	2.27 [a]
Surgical castrates	A	92.3 [b]	75.00 [ab]	6.14 [bc]	5.56 [ab]	7.92 [bc]	8.10 [ab]	
	B	92.3 [b]	75.45 [a]	6.27 [abc]	5.51 [ab]	8.85 [ab]	8.16 [ab]	
	C	91.9 [b]	75.15 [ab]	6.40 [a]	5.56 [ab]	5.93 [c]	7.61 [ab]	

[a,b,c]- LS-means with different superscripts within a column differ at $p<0.05$; TSL A = Pietrain x Large White, TSL B = pure Pietrain, TSL C= Pietrain x Duroc x Large White.

Table S2. Confidence intervals for incidence of boar taint in different meat samples.

Entire Males									
**Boar Taint**	**None**			**Strong**			Mild		
**Terminal Sire Line**	**A**	**B**	**C**	**A**	B	C	A	B	C
Heated, CI	0.67, 0.99	1.00, 1.00	0.83, 1.00	0.11, 0.44	0.00, 0.09	0.05, 0.27	0.00, 0.22	0.00, 0.09	0.00, 0.16
Microwave, CI	0.55, 0.92	0.55, 0.93	0.72, 1.00	0.05, 0.42	0.11, 0.48	0.05, 0.35	0.00, 0.26	0.00, 0.21	0.00, 0.18
Immunocastrates									
Boar taint	None			Strong			Mild		
Terminal sire line	A	B	C	A	B	C	A	B	C
Heated, CI	0.83, 1.00	0.83, 1.00	0.72, 1.00	0.05, 0.27	0.05, 0.27	0.05, 0.35	0.00, 0.16	0.00, 0.16	0.00, 0.18
Microwave, CI	0.83, 1.00	0.39, 0.82	0.67, 0.99	0.05, 0.27	0.27, 0.70	0.05, 0.38	0.00, 0.16	0.00, 0.26	0.00, 0.27
